# Call Cultures in Orang-Utans?

**DOI:** 10.1371/journal.pone.0036180

**Published:** 2012-05-07

**Authors:** Serge A. Wich, Michael Krützen, Adriano R. Lameira, Alexander Nater, Natasha Arora, Meredith L. Bastian, Ellen Meulman, Helen C. Morrogh-Bernard, S. Suci Utami Atmoko, Joko Pamungkas, Dyah Perwitasari-Farajallah, Madeleine E. Hardus, Maria van Noordwijk, Carel P. van Schaik

**Affiliations:** 1 Anthropological Institute and Museum, University of Zurich, Zurich, Switzerland; 2 Sumatran Orangutan Conservation Programme (PanEco-YEL), Medan, Sumatra, Indonesia; 3 Behavioural Biology, Utrecht University, Utrecht, The Netherlands; 4 Department of Anthropology, Boston University, Boston, United States of America; 5 University of Cambridge, Wildlife Research Group, Cambridge, United Kingdom; 6 Fakultas Biologi, Universitas Nasional, Jakarta, Indonesia; 7 Primate Research Centre, Bogor Agricultural University, Bogor, Indonesia; 8 Institute for Biodiversity and Ecosystem Dynamics, University of Amsterdam, Amsterdam, The Netherlands; Institut de Biologia Evolutiva - Universitat Pompeu Fabra, Spain

## Abstract

**Background:**

Several studies suggested great ape cultures, arguing that human cumulative culture presumably evolved from such a foundation. These focused on conspicuous behaviours, and showed rich geographic variation, which could not be attributed to known ecological or genetic differences. Although geographic variation within call types (accents) has previously been reported for orang-utans and other primate species, we examine geographic variation in the presence/absence of discrete call types (dialects). Because orang-utans have been shown to have geographic variation that is not completely explicable by genetic or ecological factors we hypothesized that this will be similar in the call domain and predict that discrete call type variation between populations will be found.

**Methodology/Principal Findings:**

We examined long-term behavioural data from five orang-utan populations and collected fecal samples for genetic analyses. We show that there is geographic variation in the presence of discrete types of calls. In exactly the same behavioural context (nest building and infant retrieval), individuals in different wild populations customarily emit either qualitatively different calls or calls in some but not in others. By comparing patterns in call-type and genetic similarity, we suggest that the observed variation is not likely to be explained by genetic or ecological differences.

**Conclusion/Significance:**

These results are consistent with the potential presence of ‘call cultures’ and suggest that wild orang-utans possess the ability to invent arbitrary calls, which spread through social learning. These findings differ substantially from those that have been reported for primates before. First, the results reported here are on dialect and not on accent. Second, this study presents cases of production learning whereas most primate studies on vocal learning were cases of contextual learning. We conclude with speculating on how these findings might assist in bridging the gap between vocal communication in non-human primates and human speech.

## Introduction

Recent studies on various species, especially primates, examined geographic variation in a wide range of behaviours to examine the presence of traditions or cultures (defined as behaviors that are common in at least one site, but are absent in at least one other site, without concomitant genetic or environmental differences among these sites [1]). Comparisons of different populations of well-studied species such as chimpanzees, orang-utans, spider monkeys and capuchin monkeys yielded a large number of behaviours that systematically varied among populations [2], [3], [4], [5]. Application of the method of exclusion (or ‘ethnographic method’) suggested that individuals acquired many of those variants through socially mediated learning rather than through environmental induction or genetic canalisation because these are excluded by statistical analyses [2], [3], [6], [7]. Recent tests that partially control for the effects of environmental and genetic differences among populations support this interpretation for orang-utans without directly demonstrating social learning (hence the absence from the definition above) [8]. Moreover, the cultural interpretation is consistent with experimental evidence for observational learning in captive great apes [9] and selective visual attention to techniques thought to be cultural among wild immatures [10], as well as with experimentally induced diffusion of behavioural alternatives through captive populations of primates [11], [12]. Taken together, it has been suggested that these studies indicate that the first hominins had a modest cultural capacity, upon which the much more elaborate cumulative technological and institutional cultures that evolved in the genus *Homo* rest (e.g. [3]).

These conclusions have been challenged (see chapters in [13]). The main point of criticism of the method of exclusion is that the method does not show evidence for social learning, which is essential to claim culture [13]. It has been argued that translocation experiments of individuals or populations would unequivocally establish social learning in the wild, but there are ethical and legal obstacles to such experiments in many species, such as great apes. As a result, some argue that the evidence for culture is stronger in fish species that great apes [14]. Alternative ways that social learning could potentially be demonstrated could be the introduction of new behaviours by a dispersing individual or when unrelated individuals living in close proximity converge upon the same behavioural variants. In addition, there is considerable debate on the impact of genetic variation on the reported behavioural dissimilarities between sites as witnessed by a recent exchange [15], [16], [17], but see [8]. At present, therefore, the evidence for primate cultures rest on plausibility.

Here we aim to advance the discussion on putative great ape culture by extending it to the vocal domain and examining genetic and ecological variation between sites. We hypothesize that like with many of the other behaviours in orang-utans, genetic and ecological variation alone cannot easily explain the reported patterns [3], [6], [7].

Our focus here is not on suggested cultural behavioural variants in great apes that improve subsistence or comfort, or serve as variations on visual and tactile social signals, but rather on qualitatively different calls, so far reported exclusively for orang-utans [3], [6], but present in other animal taxa [18], [19], [20], [21], [22], [23]. Here, we focus on different call types made during nest building (nest smacks and raspberries) or by mothers to call infants (throat scrape and harmonic uuh), and will ignore the variation in the production of the so-called kiss-squeak by using hands or leaves in addition to the lips [24]. Thus our study differs from studies that examined geographic variation (or variation among captive groups) in acoustic characteristics of the same call type (e.g. [25], [26], [27], [28], [29], [30]). These between-population differences represent accents and not dialects, which is the focus of this paper [31]. Such within-call type variation has been attributed to ecological and genetic factors, but also to vocal learning and thus argues for the existence of within-call type vocal learning in nonhuman primates [29]. Although evidence for geographic variation in discrete call types (i.e. dialects [31]) that can be attributed to vocal learning has not yet been reported for nonhuman primates, it is common in birds [18], [19], cetaceans [20], [21], [22] and some non-primate mammal species [23]. For several of these taxa the possibility of vocal cultures has been investigated [20], [21], [22], [32]. Here we examine to what extent population specificity in the presence/absence of call types can be explained by genetic and ecological factors. We find that neither of these can sufficiently explain the observed variation and that therefore a cultural explanation remains viable.

## Methods

**Figure 1:**
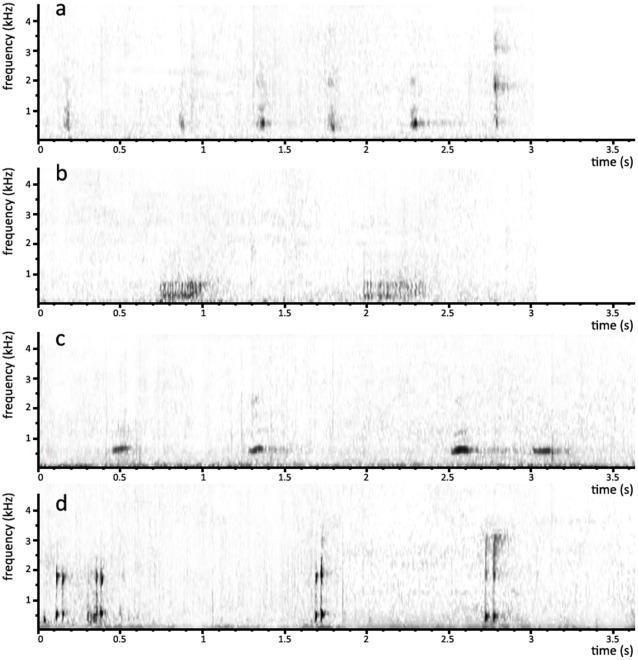
Orang-utan call spectrograms. Spectrograms of orang-utan calls: a) ‘nest smacks’; b) ‘raspberries’; c) ‘harmonic uuhs’; d) ‘throat scrapes’. The nest smack and raspberry are produced by orangutans during nest building. The harmonic uuh and throatscrape are produced by mothers towards infants that are separated from them and functions as a ‘come-hither’ call because infants return to the mother after these calls.

**Figure 2:**
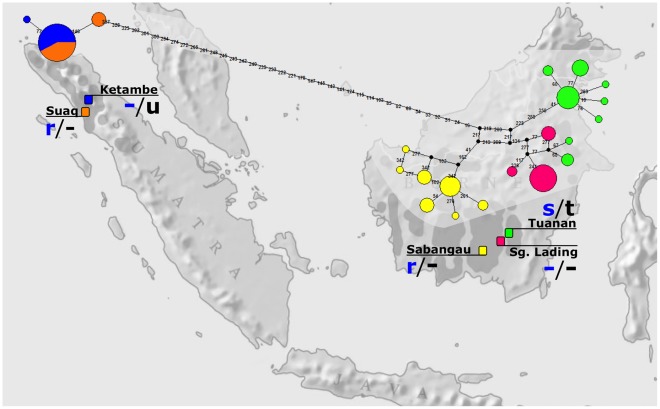
HVR-I haplotype median-joining network. A median-joining network showing HVR-I haplotypes in the different populations in relation to orang-utan calls: nesting calls and mother-infant calls. The size of each circle corresponds with the number of individuals with this particular haplotype, with the smallest circles representing one individual with this particular haplotype. Black dots indicate mutational steps connecting the sampled haplotypes, and thus represent haplotypes that were not sampled and may or may not exist. Each number on the network indicates a single base-pair mutation. First letter code in blue refers to the kind of nesting call (r = ’raspberry‘; s = ’nest smack‘; -  =  no call). Second letter code refers to the mother-infant call (u = ’harmonic uuh‘; t = ’throat scrape‘; -  =  no call).

Our analyses focused on four call types (Figure 1). Fieldwork for this study was conducted at five study sites, two on Sumatra and three on Borneo (Figure 2, Table 1). Orang-utans at all sites were well habituated to human observers. At each site orang-utans were followed from dawn to dusk and behavioural data were collected following a standardized protocol (http://www.aim.uzh.ch/orangutannetwork/FieldGuidelines.html). Researchers active at each site were focused on noting any calls the orang-utans made [33]. Many of the researchers worked at multiple sites and were familiar with the calls and behaviours at other sites. It is therefore unlikely that we report false negatives. At each site as many orang-utans as possible were followed on a regular basis and the large number of hours and numbers of individuals followed at each site minimise the probability that we report false negatives at some of the sites (Table 1). Faecal samples from orang-utans were collected at all sites and stored in ethanol or RNA later. The relevant permits for the observational work and field studies were obtained from the relevant institutes.

**Table 1:** Orang-utan site information.

Site(start of study)	Coordinates/habitat	No. hrs of focal observation	Individuals observed nesting (# making calls)	Mother-infant pairs observed (making call)	No. of sequenced individuals
Tuanan (B) (2003–)	2° 09′ S 114° 26′ E/Peat swamp	>15,000	21 (21)	8 (8)	20
Sg. Lading (B)(2005–2007)	2° 15′ S 114° 22′ E/Peat swamp	>2,000	6 (0)	4 (0)	24
Sabangau (B) (2003–)	2° 19′ S 114° 00′ E/Peat swamp	>3,000	19 (18)	4 (0)	21
Ketambe (S) (1971–)	3° 41′ N 97° 39′ E/Dryland	>15,000	20 (0)	6 (5)	16
Suaq (S) (1994–)	3° 04′ N 97° 26′ E/Peat swamp/dryland	>10,000	28 (25)	12 (0)	15

Note: (B)  =  Borneo, (S)  =  Sumatra. For the number of individuals making nests, we only included individuals that were followed for more than 10 nights, because after this number of night nests most orang-utans that occur in sites were they make nest calls were found to have made a nest call. At sites where mother-infant calls were heard, they occur once every 7.8 mother-infant follow hours for Ketambe (994 total follow hours) and 42.6 follow hours for Tuanan (5827 total follow hours). At the three sites were these calls were not heard, many more follow hours have been collected (Sabangau: 1709 hrs; Sg. Lading: 2140 hrs; and Suaq: 7665 hrs).

### Call Types and Recordings

The nesting calls examined in this study are given by all age-sex classes, except for individuals under 2–3 years of age. Calls of young individuals are made less often and are less loud and therefore we only have recordings of adult males and females. Both sexes are represented in the samples from both sites. The mother-infant calls are made by females with an infant old enough to feed or travel independently from the mother. At all sites such mother-offspring pairs were studied.

 Calls were recorded with Marantz Analogue Recorder PMD222 in combination with a Sennheiser microphone ME 64, a Sony Digital Recorder TCD-D100 in combination with a Sony microphone ECM-M907 or a Marantz PMD600 in combination with a Sennheiser microphone ME 66. Calls were digitized in Raven Interactive Sound Analysis Software (2003, Cornell Lab of Ornithology, Ithaca, NY). Because of the brief duration of throat scrapes, non-default spectrogram settings in Raven were used for comparison between harmonic uuh’s and throat scrapes in order to increase resolution and measurement accuracy in the time scale (window type  =  Hanning; spectrogram configuration: time grid spacing  = 111 samples/frame overlap  = 49.8%; frequency grid spacing  = 46.9; window size  = 221 samples; 3 dB bandwidth  = 312 Hz). Default spectrogram representation in Raven were used for comparison between nest-smacks and raspberries (window type  =  Hanning; spectrogram configuration: time grid spacing  = 256 samples/frame overlap  = 50%; frequency grid spacing  = 93.8; window size  = 512 samples; 3 dB bandwidth  = 135 Hz).

Throat scrapes are composed of 1–13 brief glottal pulses. Acoustical measurements were made on the glottal pulses. In total we measured 658 glottal pulses, 44 harmonic uuhs, 89 nest-smacks, and 35 raspberries (for the number of individuals see Table 1). Because the call types varied extensively in their acoustic structure we decided to measure four acoustic parameters that could clearly be measured from each call type and would not be influenced by recording distance so that the call types could be compared statistically. These were: duration (s), max frequency (Hz, the frequency with the maximum power (dB)), delta frequency (Hz) and max power position (%). The max power position indicates where in the call the max frequency is occurs. For example, if the max frequency of a call is exactly at half the duration of the call, the max power position will be 50%. We used a discriminant function analysis to determine whether a call could be correctly assigned to its call type based on its acoustic characteristics (cf. [27]). We conducted a separate analysis for nest smacks versus raspberries, and for throat scrapes versus harmonic uuhs. The analyses were conducted by ARL for a different manuscript (in prep) and therefore could not have been influenced by the aims of the analyses presented here. In addition, a 20% subsample of the calls was analyzed by (MEH) and similar percentages of correct assignments were found when re-running the discriminant function analyses on the subsample.

### Genetic Marker Systems

To estimate genetic distance between study sites, we utilised parts of the rapidly evolving hyper-variable segment of the mitochondrial control region (HVRI), which reflects the time since divergence from a common ancestor. Due to the rapid evolution of the HVRI region, this marker may produce homoplasy between the island populations of orang-utans, resulting in underestimation of the true genetic distance between populations. Hence, we also calculated genetic distance between sites using 1228 base pairs of three concatenated mitochondrial genes, which evolve more slowly than the HVRI and at a similar rate as coding nuclear loci.

DNA from 96 wild adult orang-utans with known provenance (Table 1) was extracted using the Qiagen stool-kit according to manufacturer’s instructions. We obtained sequence information of two mitochondrial segments in order to calculate genetic distances between orang-utan populations. First, we amplified the HVRI region, comprising part of the non-coding control region, using primers DLF 5′-CTGCCCCTGTAGTACAAATAAGTA-3′ (developed by A.N.) and D5 [34], resulting in a 357 base pair product. PCR reactions consisted of 1–40 ng template DNA, 0.25 µM of each primer, 0.2 mM dNTPs, 2 µg BSA (NEB), 2 µl of 10× PCR buffer containing 15 mM MgCl_2_, 0.6 u HotStarTaq DNA polymerase (all Qiagen), and ddH_2_O to a 20 µl-volume. Hot-start PCR reactions were carried out on a Veriti Thermal Cycler (Applied Biosystems) with the following cycling scheme: initial denaturation for 15 min at 95°C, 35–45 cycles (depending on the starting DNA concentration) of 94°C for 30 s, 58°C for 30 s and 72°C for 60 s, followed by a final extension at 72°C for 10 min. Second, we amplified a total of 1228 base-pairs (bp) from three mitochondrial genes (NADH dehydrogenase subunit 3, 345 bp; cytochrome b, 496 bp; and 16S rRNA, 387 bp), using primers developed by [35], [36]. Molarities for the PCR reactions were identical to those used in the HVR-I amplifications. Cycling conditions for all three genes were initial denaturation for 15 min at 95°C, followed by cycles of 94°C for 30 s, 58°C for 40 s and 72°C for 40 s. The PCR was finished by a final extension at 72°C for 10 min.

All PCR products were cycle-sequenced using 1 µl of PCR product, 1.75 µl 5× sequencing buffer (10 mM MgCl_2_, 400 mM Tris, pH = 9.0), 0.5 µl BigDye Terminator v3.1 (Applied Biosystems), 0.4 µM sequencing primer and ddH_2_O up to 10 µl total volume. The cycling scheme was as follows: initial denaturation at 95°C for 45 s, 30 cycles of 95°C for 30 s, 52°C for 20 s, and 60°C for 4 min. Sequencing reactions were cleaned up using 75 µl of 0.2 mM MgSO_4_, in 70% v/v EtOH. Capillary electrophoresis was performed on a 3730 DNA Analyzer (Applied Biosystems).

Complementary sequences were added to a contig and sequence identity was checked in Lasergene SeqMan Pro v7.1.0 (DNASTAR). Sequences were collapsed into unique haplotypes using Clean Collapse v.1.0.5. For HVR-I, intraspecific gene genealogies were inferred using a median joining network in Network v. 4.5.1 (available from http://www.fluxus-technology.com/). Genetic distances between pairs of populations were calculated using the software Mega v. 4.0 [37], employing the Maximum-Composite Likelihood distance with gamma parameters of 0.210 and 0.196 for the HVR-I region and the concatenated mtDNA genes, respectively.

### Statistical Analysis

The traditional hypothesis is that the call repertoire of primates has a strong genetic basis (e.g. review in [38]) and therefore that population differences in the types of calls produced among primates have a genetic basis. If there is a genetic predisposition for a particular behavioural trait, but there is no information on the genes involved, one would predict that genetic similarity between two individuals is correlated with the similarity in the trait [39]. Thus, if a genetic signal were present in for example the nesting calls, one would predict that pairs of sites sharing the same state (either presence or absence of the same call type) have a smaller genetic distance on average than pairs of sites with different states (call present in one but absent in another). To evaluate a potential genetic explanation, we applied the following Monte-Carlo procedure. We randomly redistributed the observed behaviours among the five sites a thousand times. For each randomisation, we calculated the genetic differentiation value (GDV), defined as the difference between the averaged genetic distances between the two classes of sites, as above. We carried out this analysis separately for nesting calls and mother-infant calls, using the genetic distances based on HVR-I and the mtDNA genes.

## Results

Population comparisons focused on five sites where wild orang-utans have been studied extensively (Table 1; Figure 2). Observations revealed at least two behavioural contexts in which orang-utans in different populations make very distinct sounds: nest building and infant retrieval by the mother.

All wild orang-utans build night nests on a daily basis. During the last stage of nest construction, some produce a call that varies among the five populations compared here. These variations are categorical: the raspberry and nest-smack calls are very different in their acoustic properties (Figures 1a and b). The discriminant function analysis based on the four acoustical measurements explained 100% of the variance between the two calls on the basis of one function. This function had the highest correlations with call parameters duration and maxfreq (−0.756 and −0.705 respectively). For raspberries 96.7% of the cases (n = 30) were classified correctly and for nest smacks this was 100% (n = 34). Using the leave-one out validation these percentages remained exactly the same. Thus, orang-utans in Tuanan routinely produce ‘nest smacks’ (audio file S1), and those in Suaq and Sabangau ‘raspberries’ (audio file S2). These calls have high prevalence, *i.e.* are essentially made by all individuals (Table 1), and are made on a daily basis. In contrast, such routine nesting calls are completely absent in Ketambe and Sungai Lading.

The second call concerns the maternal ‘come-hither’ call, made by orang-utan mothers just before retrieving their infant (Figures 1c–d). In three populations, all mothers examined are silent (Figure 2), but in one population, Ketambe on Sumatra, all use one call (‘harmonic uuh’: audio file S3, Figure 1c), whereas in another, Tuanan on Borneo, they use a completely different call (‘throat scrape’: audio file S4, Figure 1d). Similar to the analyses of the nesting calls, the discriminant function analysis based on the four acoustical measurements explained 100% of the variance between the harmonic uuh and the throat scrapes on the basis of one function with which call parameters duration and maxfreq had the highest loadings (−0.932 and 0.175 respectively). For harmonic uuhs 97.7% of the cases (n = 44) were classified correctly and for throat scrapes this was 100% (n = 44). Using the leave-one out validation these percentages remained very similar (95.5 and 100%, respectively). As with the nest-building calls, in populations where these calls are made they are (near)-ubiquitous in their prevalence (Table 1), and are emitted on a regular basis (albeit less than once a day).

**Figure 3:**
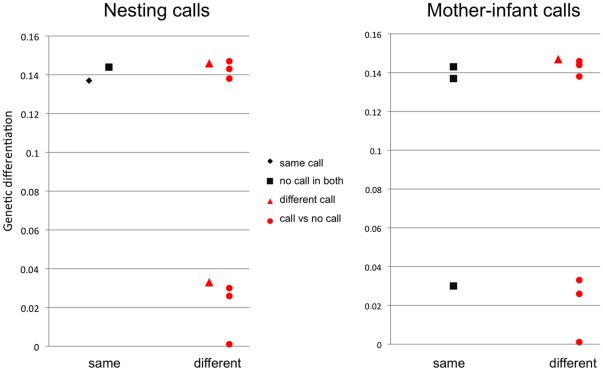
Average genetic distance between pairs of sites. Average genetic distance (maximum composite likelihood distance of HVR-I haplotypes, see Material and Methods) between pairs of sites in five orangutan populations, for two different situations: where nesting calls and mother-infant calls are the same in both sites, and where the two sites are different. If genes play a role in the production of these calls, pairs of sites with the same behavioural state should show smaller average genetic distance than pairs of sites with different behavioural states.

**Figure 4:**
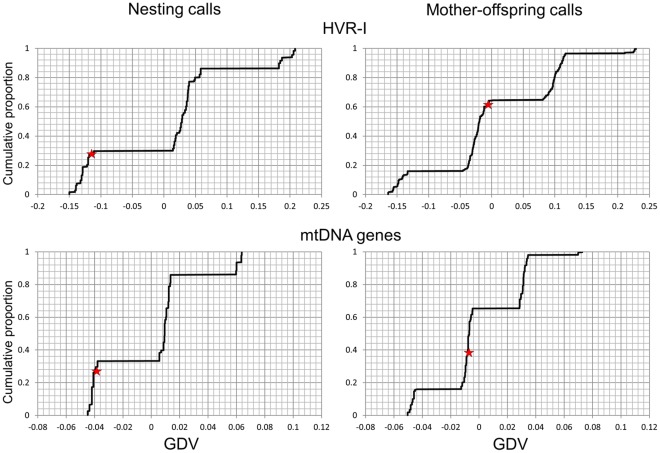
Cumulative distribution of randomised genetic differentiation values (GDV) among populations. GDVs were generated as follows: the observed behavioural states were randomly assigned to each of the 5 sites a thousand times, thereby producing site pairs with the same, but also with different behavioural states compared to those that were originally observed for each randomisation. For each randomisation, we then calculated GDV, defined as the difference between the averaged genetic maximum composite likelihood distance among sites pairs with different behavioural states and the averaged genetic maximum composite likelihood distance among site pairs with the same behavioural state. If genetic similarity played a role in the observed pattern, the observed GDVs are expected to be positive. The star indicates the value actually observed in this study.

Figure 3 suggests no relationship between average pair-wise genetic distance based on mitochondrial genes and similarities in nesting calls and mother-infant calls in the five orang-utan populations, regardless of whether we used the concatenated mitochondrial genes or the non-coding HVRI region. Indeed, if we separate these points into two classes (high and low genetic differentiation), 4 out of the 5 site-pairs that have the same behavioural state (same call or no call) are in the wrong direction (e.g. high genetic differentiation despite the same behavioural state at a site-pair). A more formal test is reported in Figure 4. The observed value is shown in relation to the cumulative distribution of the randomised genetic differentiation values. There is a trend toward lower genetic similarity between pairs of sites with the same state of the calls, opposite to prediction if the calls were genetically canalized.

Additional evidence against a purely genetic explanation is provided by the median joining network (Figure 2) which shows three of the males sampled in Tuanan as having haplotypes that were genetically closer to those found in Sungai Lading (Figure 2), where no calls are produced. Yet all males in Tuanan observed to date have been found to produce nest calls.

## Discussion

This study has shown that orang-utans produce population-specific calls that are statistically distinct in their acoustical variables and clearly constitute different call types. Hence, the study expands on an earlier study in orang-utans that showed that there was geographic variation in the orang-utan male long-distance call (the long call [25]) and other studies showing such within-call type acoustic variation for other primate species [26], [27], [28], [29]. Such within-call type geographic variation has also been suggested to be due to vocal learning and not to ecological or genetic factors [29], [40].

Here we show that population-specific calls made in the nest-building and infant retrieval contexts are independent of genetic variation across these populations, which implies that the production of these calls is at least not totally genetically canalized during development. These results are therefore in correspondence with a recent study on geographic variation in orang-utans that addressed the same question for a large number of behavioural and social variables [8]. This study found that genetic dissimilarity between populations for putative cultural behaviours was not correlated with genetic or environmental variation.

The absence of genetic effects is consistent with recent studies using data from autosomal genomes [41] and Y chromosome polymorphisms [42], which showed a surprisingly recent, not previously documented divergence time of about 400 kya and 168 kya between Sumatran and Bornean orang-utans, respectively. Hence, the similarity in patterns between and within islands suggests that these orang-utan calls are not genetically canalised. For instance, the nest raspberries appear in both a Sumatran and a Bornean site, whereas nearby sites have completely different behavioural states.

Landscape-level ecological differences (dryland vs peatswamp forest) could be excluded as potentially explaining the population specificity of these orang-utan calls because several of the differences were found in the same habitat type. All but one of the populations compared live in peat swamp habitats and those on Borneo are in close proximity (but across impassable rivers: Figure 2). They nonetheless vary greatly in either the type or the very presence of nesting calls. In addition, orang-utans at Suaq sometimes make nests in dryland forest areas and then still emit the raspberry. Ketambe, where the ‘harmonic uuh’ is produced, is a dry-land forest, whereas Tuanan, where the ‘throat scrape’ is produced, is a peat swamp. Thus not all orang-utan populations occurring in peat swamp populations make throat scrapes because these are absent in Suaq, Sg. Lading, and Sabanagau (all peat swamp sites). Similarly, nor do all orang-utan populations that occur in dryland areas produce harmonic uuh calls because part of the Suaq study site is dryland and no harmonic uuh is produced there (Table 1). In addition, even though these are different habitats, the calls are aimed at the infant, which is rarely more than a few meters away in the canopy. This patterning among habitats is therefore consistent with the observation that call propagation properties of different habitats become apparent only at much larger distances than observed here [43], and that habitat differences have been used in primate studies to explain gradual changes of the same call types, such as subtle frequency changes, but not for the replacement of one call type by another [26], [27], [28].

The potential role of possible small-scale ecological variation between sites was not addressed and its potential influence on acoustic signals deserves more study. However, for various reasons we think that potential variation of small-scale ecological variation (e.g. leaf density, canopy structure) at best has a limited impact on our results. First, variation in habitat ecology in general is often considered to have an influence on the acoustic structure of long-distance signals, not of short-distance signals [43], [44]. Second, the three sites on Borneo are all in the same peat-swamp forest block and consequently these sites are ecologically very similar [45], [46], in which orang-utans show many similarities in foraging and nesting behaviour [47]. Thus, it is unclear why we find completely different calls types rather than subtle differences within call types. Indeed, studies focusing on bird song that have described variation in bird song have mainly found differences between very different habitats such as open and closed ones within the same species [18], [48], [49], and where within-species dialects have been reported vocal learning has been the predominant explanation, with ecology or genetics given much less prominence (e.g. [50]). Finally, the influence of potential between-population variation in sound propagation for these calls is probably very limited because excess attenuation differences for the frequency range of the calls studied here are most pronounced below heights of 1m above the ground rather than at the much greater heights [44], [51] at which orang-utans in these forests build nests and forage [47].

It could also be argued that differences in sociality affect our results, because Sumatran orang-utans are more social than those on Borneo [47]. The relevance of overall sociality variation on nest calls is probably negligible, however. First, orang-utans often nest solitarily (or in the case of mothers with offspring, only with their offspring) and for Tuanan it has been shown that the presence of associates does not affect the production of the nesting calls [52]. Second, the influence of overall sociality on mother-infant calls is likely to be limited because the three Bornean populations show a similar sociality, but nevertheless vary in the presence or absence of mother-infant calls. The same argument holds for the two Sumatran populations. They also show similar sociality, but a mother-infant call is found in only one of these populations.

The calls had high prevalence where they occurred, basically being made by all relevant individuals (Table 1). Thus, the results presented here strongly suggest that these sounds were invented in each population and subsequently spread through social learning (cf. [33]). This interpretation is consistent with evidence in orang-utans for the two critical elements for culture: innovation and social learning. First, fieldworkers have observed that individual apes sometimes produce ‘private’ calls (i.e. calls that are unique to this individual) in play or nest building (unpublished obs., M. van Noordwijk, M. Bastian, M. Paul), suggesting that the invention of novel calls is not implausible. Second, studies show that captive orang-utans and chimpanzees can socially learn to give calls that are not part of the species-specific repertoire, such as a whistle, and subsequently show flexible usage of such calls [53], [54], [55]. Outside the call domain strong indirect evidence for social learning has been found in a number of orangutan field studies [10], [45], [56]. Taken together, the recent wild studies [3], [6], [24] and captive studies [53], [54], [55] on great apes have recently been interpreted to indicate that great apes have some voluntary control over respiration and vocal fold adduction [38]. It is therefore perhaps no coincidence that the signals recorded in this study are calls that have either no (raspberry and nest smack) or little involvement of the vocal folds (throat scrape and harmonic uuh), with only the latter two showing higher harmonics (even though not clearly depicted for harmonic uuhs in figure 1).

Several types of vocal learning have been described [57] and it is therefore relevant to determine which type of vocal learning could be important for the results presented in this paper. The calls investigated in this study are distinct from other calls types in the orang-utan’s call repertoire [33] and not examples of calls from the existing repertoire used in novel context in some populations. Therefore the results presented here are not an example of contextual learning [57], but are likely to have been innovated in the populations were they are found and thus an example of production learning.

It has been suggested that there is a large gap between the vocal communication of nonhuman primates and human language, making it hard to see how the latter could have evolved from the former (review in [58]). However, the presence of these cultural calls in orang-utans suggests the gap is perhaps not as wide as often perceived. Orang-utans occasionally invent calls with an arbitrary acoustic structure. The spread of these calls can be understood through shared need, so the audience could easily grasp from the context what the function of the calls should be (although the function of the nest calls remains unknown), in a process very similar to the social learning of the functional use of innate vocalizations in other species (e.g. [59], [60]). Thus, the orang-utan findings imply that we are dealing with arbitrary symbols that had acquired a shared meaning – two important elements of language.

## Supporting Information

Audio File S1 **‘Nest-smack’ nest call from Tuanan. Nest smacks at 0.1 s, 0.7 s, 1.4 s, 1.9 s, 2.3 s, 2.8 s and 3.3 s.**
(WAV)

Audio File S2 **‘Raspberry’ nest call from Sabangau. Raspberries at 0.8 s and 3.0 s.**
(WAV)

Audio File S3 **‘Harmonic-uuh’ mother-infant vocalization from Ketambe. Harmonic uuh at 0.4 s.**
(WAV)

Audio File S4 **‘Throat scrape’ mother-infant call from Tuanan. Throatscrapes at 0.1 s, 0.3 s, 1.7 s, 2.7 s, 3.9 s, 4.4 s and 5.2 s.**
(WAV)
